# Variation in structural location and amino acid conservation of functional sites in protein domain families

**DOI:** 10.1186/1471-2105-6-210

**Published:** 2005-08-25

**Authors:** Birgit Pils, Richard R Copley, Jörg Schultz

**Affiliations:** 1 Department of Bioinformatics, University of Würzburg, Biocenter, Am Hubland, 97074 Würzburg, Germany; 2 Wellcome Trust Centre for Human Genetics, University of Oxford, Headington, OX3 7BN Oxford, UK

## Abstract

**Background:**

The functional sites of a protein present important information for determining its cellular function and are fundamental in drug design. Accordingly, accurate methods for the prediction of functional sites are of immense value. Most available methods are based on a set of homologous sequences and structural or evolutionary information, and assume that functional sites are more conserved than the average. In the analysis presented here, we have investigated the conservation of location and type of amino acids at functional sites, and compared the behaviour of functional sites between different protein domains.

**Results:**

Functional sites were extracted from experimentally determined structural complexes from the Protein Data Bank harbouring a conserved protein domain from the SMART database. In general, functional (i.e. interacting) sites whose location is more highly conserved are also more conserved in their type of amino acid. However, even highly conserved functional sites can present a wide spectrum of amino acids. The degree of conservation strongly depends on the function of the protein domain and ranges from highly conserved in location and amino acid to very variable. Differentiation by binding partner shows that ion binding sites tend to be more conserved than functional sites binding peptides or nucleotides.

**Conclusion:**

The results gained by this analysis will help improve the accuracy of functional site prediction and facilitate the characterization of unknown protein sequences.

## Background

Protein function is determined by the spatial configuration and type of amino acids at functional sites. Knowledge of functional sites provides valuable information for the assignment of molecular function, potential physiological binding partners and hence drug design. Tasks performed by functional sites range from the binding of small molecules like ions, cofactors, metabolic substrates or high molecular weight compounds such as nucleic acids and peptide chains, to catalysing chemical reactions in the active centre of enzymes.

The exponentially growing number of uncharacterised protein sequences in the public databases has turned the development of automatic identification of functional sites into an important research field and many computational methods focusing on this area have been described in recent years (for review see [1-3]). In contrast to structural approaches that search for ligand binding pockets on the protein surface using molecular modelling [4,5], network analysis [6], or compare the protein surface to structures with known interacting sites [7,8], many methods are based on a set of homologous sequences combined with evolutionary or structural information. The evolutionary trace (ET) method [9,10], for example, searches for a structural cluster of conserved residues. Beginning with a sequence identity tree from a set of homologous proteins, the tree is scanned for subgroup-specific residues, which are invariant within the subgroup but vary between subgroups. These residues, called evolutionary trace residues, and the residues that are invariant in all sequences are then mapped onto a representative 3D structure and clusters of high ranking residues, corresponding to the inner nodes of the tree, are searched. These clusters usually coincide with the functional center of the protein. Improvements of the ET method use sequence weights based on their similarity (weighted evolutionary tracing) and an amino acid substitution matrix to account for biochemically similar amino acids in the identification of the trace residues [11], they consider the evolutionary distance between proteins due to the phylogenetically biased databases [12] or allow different rates of amino acid substitutions at protein sites [13]. A similar approach is focusing more on structural information and calculates a conservation score at each position under consideration of the behaviour of spatial neighbours [14].

Most of the above mentioned methods assume that functional sites are under high selective pressure and conserved within the protein, so that functional sites can easily be detected by lower rates of amino acid substitutions. However, functional sites can vary in subfamilies and homologous protein sequences can perform different functions using a different set of functional residues. Accordingly, interaction interfaces can vary in their location in distant homologues and this has to be considered if interaction interfaces are inferred from homologous proteins [15,16].

Prior to their prediction, it is necessary to understand the arrangement and properties of functional sites, as well as how protein families and single sequences differ in their use. Effort towards this direction has been made by several groups, who studied physicochemical properties of protein-protein interaction interfaces found in homodimer, heterodimer or intra-chain domain complexes [17-21], as well as protein-DNA interaction interfaces [22].

Here, we perform a large-scale analysis of functional sites extracted from experimentally determined protein-ligand complexes stored in the protein data bank (PDB) [23], grouped by the presence of conserved protein domains described in the SMART database [24]. The classification into protein families enables us to find differences in amino acid conservation and use of specific locations for functional sites between the families. The analysis shows that domains vary strongly in the conservation of interacting sites and provides useful information for the prediction of interacting sites based on homologous protein sequences.

## Results and discussion

Our analysis of interacting sites is based on conserved protein domains found in the structures of the protein data bank (PDB). Family sequence alignments of protein domains were retrieved from the SMART database and used to scan the protein sequences of the PDB with domain-specific Hidden Markov Models (HMMs). Wherever a domain was identified, all interactions between an amino acid belonging to this domain and any ligand were extracted and the position of the interacting amino acid was transferred onto the HMM consensus.

Table 1 lists the number of sequences with ligand interactions extracted from PDB and the number of interacting sites found in these sequences [see Additional file 1]. The HMM consensus was used as a reference sequence to be able to compare the location of interactions among different sequences of a domain family. The positions in the consensus sequence correspond to positions shared by most sequences of the domain family (match states) and most domains interact only in regions for which an HMM match state exists. In contrast, if the interacting position corresponds to an insert state in the alignment to the HMM consensus, the information of interaction could not be transferred to the domain consensus. There are several domains, which have a comparatively large number of interacting sites located in loop regions. These amino acid sites are only present in subfamilies of the domain and are lacking an HMM match state, so that mapping the information of interaction onto the HMM was not possible. An overview of the number of interactions at HMM insert states for all investigated protein domains is given in table 1 [see Additional file 1]. In domains of the immunological system, e.g. the immunoglobulin (IG) and immunoglobulin V type (IGv) domains, up to 50% of the interacting sites are located in these regions. Strikingly, other extra-cellular domains (fibronectin type 3 (FN3), C-type lectin (CLECT), leucine rich repeat C-terminal domain (LRRCT)) also present a large number of interacting sites in loop regions. These domains all have a common involvement in highly specific recognition processes, where any restrictions in the choice of amino acid or structural constraints would be disadvantageous for the function of the domain. The loop regions without any match states exactly fulfil this condition and therefore biochemical properties of the domain can be fine-tuned to complement the appearance of the ligand. The increased use of variable loop regions for functional sites seems to be a common characteristic of extracellular highly ligand-specific domains.

### Conservation of location of interacting sites

In order to compare the use of specific interacting positions within a domain family we introduced a score (ConsInt) to measure the conservation of interaction at a given site in the family alignment. The score reflects the importance of an amino acid site in domain interactions and ranges between 0 and 1. Scores greater than 0 mean that at least two non-identical sequences interact at this position, and a score of 1 arises if all sequences interact at this position. For the comparison of the interaction scores and amino acid conservation, we only calculated the score for sites, where the interaction is achieved by atoms of the amino acid side chain. These scores are generally smaller than those calculated for side chain and backbone interactions, because side chain interactions are only part of the total interactions and many sites are very flexible in the contribution of atoms to the interaction interface. Corresponding positions in homologous sequences can interact with side chain atoms in one sequence and exclusively with backbone atoms in the next sequence.

**Figure 1:**
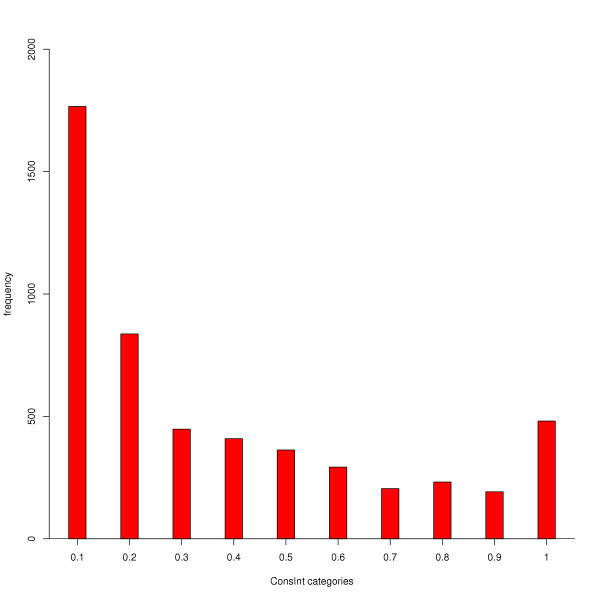
**Distribution of interaction scores**. The interaction score reflects the importance of a functional sites in establishing an interaction. Surprisingly, only few interacting sites are absolutely conserved in their location within the whole protein family and characterized by high interaction scores. The majority of interacting sites feature small interaction scores. This shows that these sites are only used by a few sequences of the domain family for establishing an interaction, which can also be caused by the different nature of ligands.

**Figure 2:**
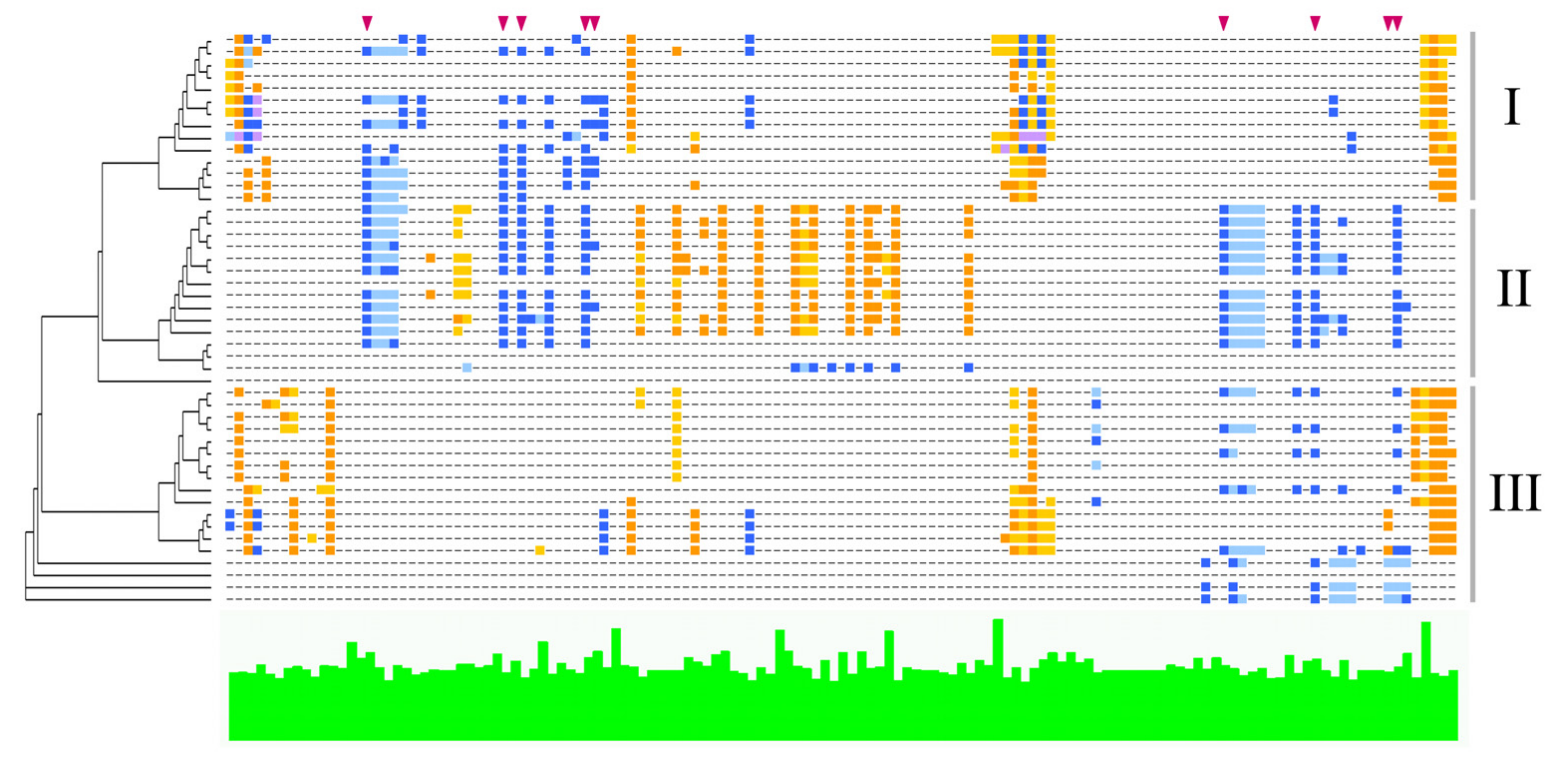
**Interaction profile of the RICIN domain**. Alignment of positions corresponding to an HMM match state. Sites interacting with saccharides are indicated in blue, peptide interactions in orange, and sites interacting with both ligands, saccharides and peptides, are indicated in purple. Light colours represent backbone interactions, darker colours involve side chain atoms. The amino acid conservation is visualized by green bars below the alignment. Sugar binding sites described in the literature are indicated by red arrows above the alignment [41]. Several positions (1, 3, 4, 22, 42, 58, 88, 90, 122) are located in the vicinity of a glycosylation site, but do not specifically interact with saccharides. The unrooted tree reflects the classification into three main subgroups with different interaction sites. Group II harbours two sugar-binding sites, group I and III originate from tandem RICIN domains, in which group I preserved the N-terminal sugar-binding site and group III the carboxy-terminal binding site. PDB identifiers from top to bottom: 1PC8 (B: 5–131), 1TFM (B: 5–131), 2MLL (B: 5–131), 1CE7 (B: 5–131), 1ONK (B: 9–135), 1PUM (B: 9–135), 1M2T (B: 257–383), 1OQL (B:13–139), 1ABR (B: 13–139), 2AAI (B: 8–134), 1HWO (B: 10–135), 1HWP (B: 10–135), 1HWN (B:10–135), 1HWM (B:3–266), 1V6U (A: 312–436), 1ISW (A:312–436), 1ISV (A:312–436), 1ITO (A:312–436), 1V6W (A: 312–436), 1V6X (A: 312–436), 1XYF (A:312–436), 1ISY (A: 312–436), 1ISZ (A:312–436), 1V6V (A:312–436), 1ISX (A:312–436), 1KNM (A:7–131), 1KNL (A:9–133), 1BFM1MC9(A:9–133), 1QXM (A: 29–157), 1PUM (B: 140–262), 1M2T (B: 390–510), 1ONK (B: 140–262), 1OQL (B: 140–262), 1PC8 (B: 136–254), 1TFM (B: 136–254), 2MLL (B: 136–254), 1CE7 (B:136–254), 2AAI (B: 138–261), 1ABR (B: 143–266), 1HWO (B: 138–262), 1HWP (B: 138–262), 1HWM (B: 138–262), 1HWN (B: 139–263), 1FWU (A: 3–123), 1DQG (A: 4–124), 1DQO (A: 4–124), 1FWV (A: 3–123)

The distribution of interaction scores is shown in figure 1. It is remarkable that there are only a few positions in all of the investigated domains with the maximum interaction score. Smaller interaction scores can emerge from the use of ligands with distinct functions in the PDB data, for example, if a domain is in complex with its native substrate or another time with a regulatory protein that binds to a remote part of the domain. Absence of the substrate in the latter case leads to lower interaction scores for important functional sites: even medium interaction scores can indicate significant interactions. A large proportion of amino acid sites have very low interaction scores, presenting sites that are only occasionally involved in interactions or that are specific to a subgroup. Subgroups tend to use the same functional positions, while the functional sites can vary between subgroups. This behaviour is also reflected in the distance trees of the domains. The carbohydrate binding RICIN domain in figure 2 exemplifies the divergence of functional sites within a domain family. By inspecting the arrangement of functional sites in the different RICIN sequences, the RICIN family can be divided into three main subgroups of distinct functions. While one subgroup possesses two carbohydrate binding sites and a peptide binding region (group II in figure 2), one subgroup is limited to the N-terminal carbohydrate binding region (group I) and one to the C-terminal carbohydrate binding region (group III). Absence of a carbohydrate binding site in the first or third group is unlikely to be an artefact of the crystallization process, since the domains were crystallized in complex with an adjacent sugar bound domain. The classification observed by the interaction profile is also reflected in the cladogram given to the left of the interaction profile. Interestingly, the subgroups that preserved only one of the carbohydrate binding sites originate from proteins with tandem RICIN domains, which arose from gene duplications [25], so that the proteins are again provided with two carbohydrate binding sites. A single RICIN domain can be divided into three subunits of approximately 40 amino acids in lengths that have evolved from an ancient galactose binding peptide [26]. These subunits represent the differently specialized binding sites in the domain family. In proteins carrying two RICIN domains, only the first subunit of the first RICIN domain and the last subunit of the second RICIN domain preserved their ability to bind carbohydrates, while one subunit has specialized on binding peptides (subgroup II in figure 2). The RICIN domain is a good example how homologous sequences belonging to different subfamilies of a domain specialized on binding different ligands and thus, on various functions. The functional sites are no longer conserved throughout the whole family and they could not be inferred from homologous sequences. However, functional sites can be predicted, if the information is taken from the most closely related sequences.

**Figure 3:**
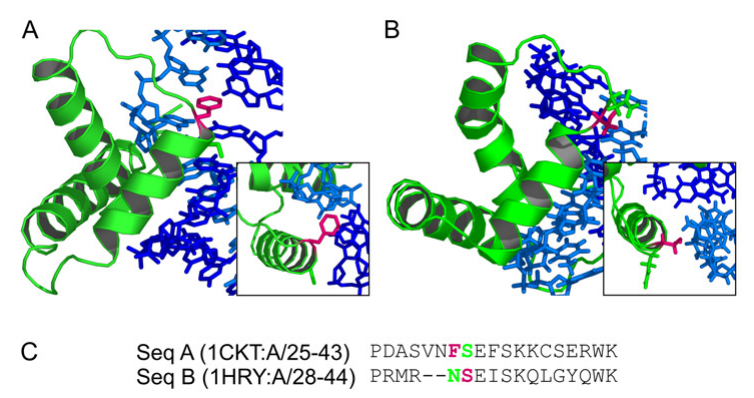
**Variable location of interacting amino acid residues in the HMG domain**. Sequence specific interaction by the high mobility group (SMART: HMG) domain (green) is achieved by an amino acid side chain (pink) pointing into the DNA double helix (blue). The interaction is achieved by a phenylalanine in figure 3a [28] or by a serine residue in 3b [27]. The sequence alignment (figure 3c) reveals that these two interacting residues are not located at corresponding position.

Alternating locations of functional sites are especially prominent in DNA binding domains, like the C2H2-zinc finder and homeodomains [22]. Another example is the high mobility group (HMG) domain, which is shown in complex with its target DNA in figure 3. Here, an amino acid side chain pointing into the DNA helix recognizes the DNA bases of the target sequence. This key position is located in the loop connecting the first and second alpha helix of the domain. The contact is carried out by a serine residue in figure 3a (pdb id: 1hry[27]). In contrast, a phenylalanine establishes the contact in figure 3b (pdb id: 1ckt[28]) and the serine residue corresponding to the structure shown in 3a is pointing away from the DNA helix. In the sequence alignment, these two key positions are located adjacent to each other. Many other domains show this variability in the location of interacting amino acid residues and profit from the flexibility to fine-tune substrate specific binding sites based on the same structurally conserved protein fold.

### Amino acid conservation of interacting sites

**Figure 4:**
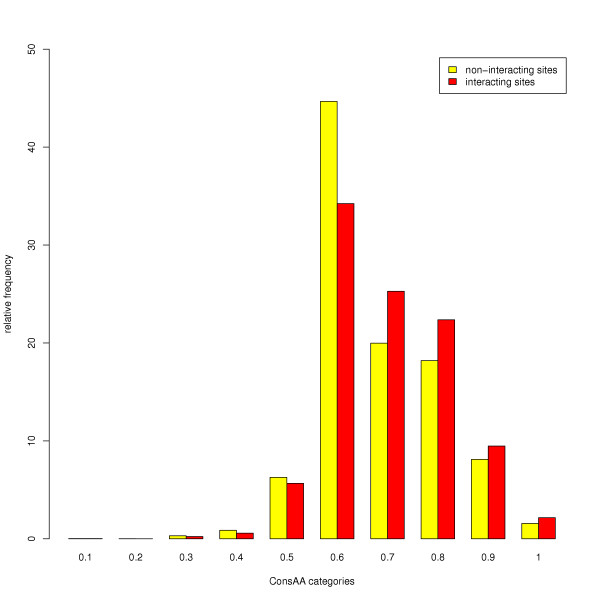
**Amino acid conservation of interacting and non-interacting sites**. Non-interacting sites (yellow) are slightly more highly conserved than interacting sites (red) as shown by the shift to higher amino acid conservation of interacting sites.

Having observed great differences in the location of interacting sites within conserved domains, the question arises how these sites behave with regard to their amino acid conservation. It is generally believed that interacting sites are more highly conserved than non-interacting solvent-assessable sites. However, about one third of all interactions in our analysis were achieved by backbone atoms only, so that the kind of amino acid has no direct effect on the interaction. For the remaining two thirds, which account for side chain interactions, we calculated a score for the conservation of amino acid similar to the interaction score ConsInt. We next compared the relative frequency distribution of the amino acid conservation score of interacting sites with the one of non-interacting sites, which are composed of amino acids located in the core of the domain as well as non-interacting surface residues. The distribution of interacting sites is slightly shifted to higher amino acid conservation scores (figure 4). This shift is clearly visible and is statistically significant (Kolmogorov-Smirnoff test: p-value < 2, 2 ×10-16), although the data have not been restricted to surface residues. Our finding that interacting sites are more highly conserved, on average, is in accordance with the results of other groups who reported that domain-domain interfaces are better conserved than the rest of the surface residues [29].

**Figure 5:**
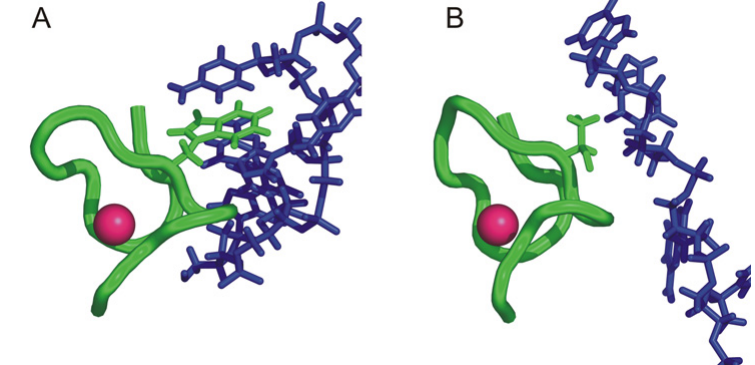
**Substrate specific interaction by varying the type of amino acid**. Substrate specificity in the zinc finger domain (SMART: ZnF_C2H2) is ensured by various amino acids that interact with the bases of the DNA. The protein domain is highlighted in green, the DNA chain in orange and the zinc atom in red.

Strikingly, there are many interacting sites with very low amino acid conservation scores. A possible explanation could be that very few residues of the interaction interface are important for a stable interaction and conserved in their amino acid. It has been shown that only a subset of interface residues contribute a crucial part to the binding energy, while residues around these so-called hot spots are less conserved [30,31]. Another explanation could be the increased specificity for various ligands. The data set contains orthologous sequences, which might be conserved in function and substrate specificity and paralogous sequences, which might have accumulated mutations throughout evolution and adopted new substrate specificity. The zinc finger domain, for example, interacts mainly through an aromatic residue with the nucleic acid, but specificity is provided by a nearby position, which can vary in its amino acid and is also interacting with the bases of the nucleic acid (figure 5). Hence, the variety of amino acids at functional sites could be advantageous to recognize numerous different ligands by the same domain family.

### Correlation of interaction and amino acid conservation

**Figure 6:**
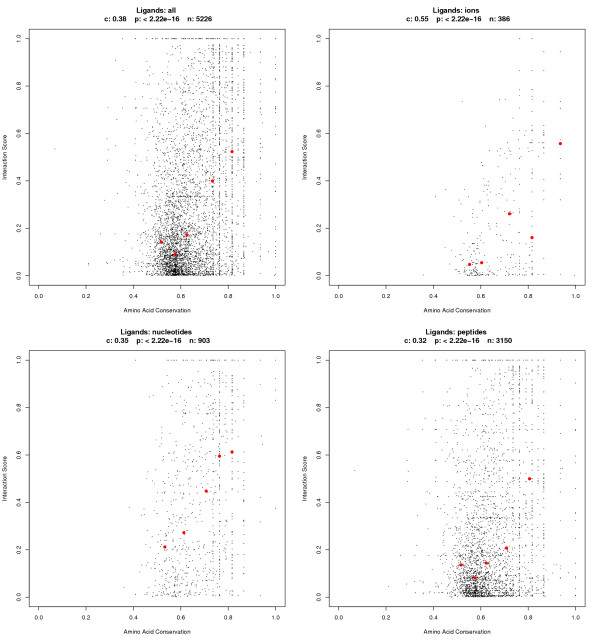
**Correlation of interaction scores and amino acid conservation**. For better visualization of the correlation, the data was divided into five groups corresponding to the 0–20% quantile, 20–40% quantile, etc. of the amino acid conservation scores and then the median interaction score and median amino acid conservation score was calculated for each group and plotted with red dots. The correlation coefficient, p-value and population are indicated for each ligand above the graphs. The correlation coefficient was calculated according to Pearson's method under the null-hypothesis of no correlation (c = 0).

In order to test whether often-used interacting sites coincide with highly conserved sites we plotted the interaction score against the amino acid conservation score (figure 6). Since our dataset includes various ligands, which might have different preferences in terms of the amino acid conservation or interaction, we divided the data by the type of ligand. Groups analyzed correspond to peptides, nucleotides, ions and all ligands, including those, which could not be classified into one of the prior groups. Statistical analysis detected a significant positive correlation between the interaction score and its amino acid conservation in all observed groups (see figure 6 for details). The trend to higher interaction scores with increasing amino acid conservation is clearly visible if the data are divided into groups and the median calculated for each group. The median values of ion binding sites are shifted to higher amino acid conservation scores compared to the other three ligand groups, indicating that ion binding sites are more highly conserved than sites binding peptides, nucleotides or other small molecules. The highest median interaction scores are found in the group of nucleotide ligands, consequently nucleotide binding sites preferentially use the equivalent positions in homologous sequences. Interestingly, sites interacting with peptides are not less conserved in their amino acids but are more flexible in the location of interaction compared to nucleotides. An unexpected finding is the great variance of amino acid conservation scores for high interaction scores, especially in the nucleotide and peptide group. This indicates that these interacting sites are very flexible in the type of amino acid and specialized to complement the ligand and to increase specificity of the protein-ligand interaction.

## Conclusion

Our analysis reveals that functional sites can be highly variable in their amino acid conservation and very flexible in using various locations in the protein domain. The properties of functional sites are dependent on the protein family and can vary from highly conserved, as observed in enzymes involved in DNA replication, to protein families that are highly variable with various amino acids at various locations, as for example immunoglobulins or carbohydrate-binding domains. Similar results were obtained by other groups. Pachenko and co-workers analyzed 86 domains from the CDD database and report that functional sites of homologous sequences can greatly differ in their physicochemical properties and their location in the three-dimensional structure [32]. Variability in functional sites was also described by Devos and Valencia. By comparing the conservation of binding sites in structural alignments, they found high conservation in diverged sequences contrasting highly similar sequences with different interacting sites [33]. Our findings present valuable information for the improvement of methods to predict functional sites. In most of these methods, the prediction is based on a set of homologous sequences. This approach results only in reliable predictions if the investigated protein family is conserved in most of the functional sites. Approaches to improve the accuracy of prediction have been made recently by using orthologous proteins with presumably the same ligand specificity [34] or by sub-typing protein families [35]. With the growing number of sequences within protein families, trends that consider variability of functional sites and use subgrouping aided by experimental information might become widely accepted in the future and promise to be successful in the prediction of functional sites.

## Methods

### Data set

The analysis is based on the October version of PDB (27969 structures). All protein sequences extracted from PDB files were scanned against all SMART domains (667) using hmmsearch from the HMMER package (version 2.3.2) [36]. Profile HMMs were retrieved from the SMART family alignments and score thresholds were used as assigned by SMART for each individual domain [37]. The search resulted in 8747 protein structures containing at least one of 480 SMART domains. For those structures containing a protein-ligand interaction, we calculated the distance for each atom of all protein compounds to each atom of all ligands. We considered amino acids as interacting if the distance of any atom of the amino acid to any atom of the ligand was smaller than 4 Angstrom, which is a very conservative threshold and is in the range of the two oxygen atoms in a hydrogen bond. Interactions to water molecules were neglected, as well as interactions between identical chains, because homodimers can present artefacts arising from the crystallization process. We are aware of losing information about naturally occurring homodimers. If more than one model of the protein structure exists, for example in the case of NMR data, all models were taken into account and a position was treated as interacting if more than 50% of all models harbour an interaction at this position. For each domain family, a multiple sequence alignment was generated from the sequences identified in the PDB scan for SMART domains. The alignments were created with hmmalign (HMMER package), according to the SMART profile HMMs. Distance trees were created with PROTDIST and FITCH, both from the Phylip package [38].

A problem with using the protein data bank is the overrepresentation of some proteins, while others are completely absent. To deal with the biased nature of the database, we used sequence weights, correlated to the evolutionary distance between the sequences. The distance is small for similar sequences, so that these proteins are weighted with a negligible small factor. Although many proteins are redundant in our dataset it is advantageous to consider all proteins because they can be bound to different ligands or the complex crystallized under different conditions. The most important interacting sites should clearly stand out in the large scale analysis.

Ligands interacting with protein domains were, wherever possible, classified into groups of peptides, nucleotides or ions. The group of ions was restricted to small ions and excluded typical buffer anions. The groups of peptides and nucleotides also included modified molecules that are functionally alike. We also analysed all ligands together, including carbohydrates, buffer ions and other small molecules.

### Calculation of scores

For each alignment position consistent with the HMM consensus sequence, we calculated a position-specific interaction conservation score (ConsInt) to describe how well a position is conserved in ligand interactions within the domain family.





where N is the number of sequences in the alignment, Dist(seq_a_, seq_b_) is the phylogenetic distance between sequence a and b obtained from the phylogenetic tree and Int_i _takes a value of 1 if sequence a and b both interact at position i, and 0 otherwise. The score ranges from 0 to 1 and is 1 if all sequences interact at the position of interest. To obtain a score greater than 0 for a certain position at least two non-identical sequences have to interact at this position. In this way, interactions to non-physiological ligands like artificially synthesized peptides should be lost, and important interacting positions should have noticeably higher scores. The score takes into account the phylogenetic distances between the sequences so that highly similar sequences are weighted more weakly, in contrast to interacting sites in divergent sequences, which are weighted more strongly.

Similar to the interaction score, a position specific amino acid conservation score (ConsAA) was calculated.



Here, Subst_i_(a, b) measures the similarity between the amino acids at position i in sequences a and b. It is based on the VTML 160 substitution matrix [39]. The conservation score was normalized between 0 (low aa conservation) and 1 (high aa conservation) ranging from the lowest to the highest score of the amino acid substitution matrix. It is important to note that our amino acid conservation scores do not describe protein families as found in the SMART database, but only the data set used in our analysis, so that the amino acid conservation score can be compared with the interaction conservation score for each position in the family alignment.

The software suite R was used for the statistical analyses [40]. The concentration of data points on vertical lines found for higher amino acid conservation scores in the scatter plots of figure 6 are an effect of the discrete values of the amino acid substitution matrix and the few substitutions at conserved sites.

## Authors' contributions

BP performed the analysis and prepared a draft of the manuscript. RRC contributed to the generation of the data set and JS supervised the project. All authors were involved in the design of the project and the refinement of the manuscript.

## Supplementary Material

Additional file 1 **Table 1 – Interacting sites in protein domains **For each domain, the number of domains with ligand interactions extracted from PDB (# PDB), the number of interacting sites corresponding to HMM match states (int sites), the number of interacting sites corresponding to HMM insert states (loop sites), the percentage of insert state interacting sites from the total number of interacting sites (% loop) and the number of interacting sites in the domain consensus (domain int sites) are given. The last column considers conserved positions in the domain consensus sequence, while all other columns count sites in sequences belonging to the domain family. The number of conserved interacting sites (domain int sites) can be 0 despite plenty of interacting sites in family sequences if the site-specific interaction score does not yield a positive value due to identical sequences. Abbreviations of domain names are according to SMART [24]. Click here for file
